# Macrophage Death as a Pharmacological Target in Atherosclerosis

**DOI:** 10.3389/fphar.2019.00306

**Published:** 2019-04-04

**Authors:** Wim Martinet, Isabelle Coornaert, Pauline Puylaert, Guido R. Y. De Meyer

**Affiliations:** Laboratory of Physiopharmacology, University of Antwerp, Antwerp, Belgium

**Keywords:** cell death, macrophages, atherosclerosis, apoptosis, necroptosis, pyroptosis, ferroptosis, autophagy

## Abstract

Atherosclerosis is a chronic inflammatory disorder characterized by the gradual build-up of plaques within the vessel wall of middle-sized and large arteries. Over the past decades, treatment of atherosclerosis mainly focused on lowering lipid levels, which can be accomplished by the use of statins. However, some patients do not respond sufficiently to statin therapy and therefore still have a residual cardiovascular risk. This issue highlights the need for novel therapeutic strategies. As macrophages are implicated in all stages of atherosclerotic lesion development, they represent an important alternative drug target. A variety of anti-inflammatory strategies have recently emerged to treat or prevent atherosclerosis. Here, we review the canonical mechanisms of macrophage death and their impact on atherogenesis and plaque stability. Macrophage death is a prominent feature of advanced plaques and is a major contributor to necrotic core formation and plaque destabilization. Mechanisms of macrophage death in atherosclerosis include apoptosis, passive or accidental necrosis as well as secondary necrosis, a type of death that typically occurs when apoptotic cells are insufficiently cleared by neighboring cells via a phagocytic process termed efferocytosis. In addition, less-well characterized types of regulated necrosis in macrophages such as necroptosis, pyroptosis, ferroptosis, and parthanatos may occur in advanced plaques and are also discussed. Autophagy in plaque macrophages is an important survival pathway that protects against cell death, yet massive stimulation of autophagy promotes another type of death, usually referred to as autosis. Multiple lines of evidence indicate that a better insight into the different mechanisms of macrophage death, and how they mutually interact, will provide novel pharmacological strategies to resolve atherosclerosis and stabilize vulnerable, rupture-prone plaques.

## Introduction

Atherosclerosis is a progressive inflammatory disease of large- and medium-sized arteries that may start as early as childhood (Lusis, 2000). Due to endothelial dysfunction, low density lipoprotein (LDL) particles are able to accumulate in the intima of the vessel wall where they are prone to a wide range of chemical alterations. Modification of LDL into oxidized (ox) LDL activates endothelial cells (ECs) to enhance the expression of various cell adhesion molecules such as cell adhesion molecule 1 (VCAM-1) and intercellular adhesion molecule 1 (ICAM-1), thereby mediating infiltration of inflammatory cells such as monocytes and lymphocytes. Together with the proliferation of vascular smooth muscle cells (VSMCs), lipid deposition and matrix accumulation, vascular inflammation (especially monocytes) drives the build-up of an atherosclerotic plaque (Moore and Tabas, 2011). Indeed, once trapped in the intima, monocytes undergo differentiation into macrophages and turn into foam cells by the uptake of modified LDL. Formation of macrophage foam cells in the intima is a major hallmark of early stage atherosclerotic lesions (Chistiakov et al., 2017). Interestingly, plaque macrophages are very heterogeneous, displaying a variety of subtypes depending on their protein expression patterns and activation stimuli (Wilson, 2010). As a consequence, they exert either beneficial or harmful functions in atherosclerosis. On the one hand, macrophages are capable of scavenging cytotoxic lipoproteins and other harmful substances in the plaque such as dead cells, especially in the early stages of atherogenesis, to avoid cytotoxicity within the developing lesion (Schrijvers et al., 2007). Efficient clearance of dead cells is essential for preventing secondary necrosis and also triggers an anti-inflammatory response through the release of anti-inflammatory cytokines. Moreover, macrophages can enhance tissue repair by promoting extracellular matrix synthesis and VSMC proliferation, which in turn enhances plaque stability. On the other hand, advanced plaques contain large numbers of macrophages with a pro-inflammatory phenotype that secrete matrix degrading enzymes, thereby contributing to plaque destabilization, plaque rupture and thrombotic events. Macrophages in advanced plaques also contribute to death of surrounding cells either by releasing toxic oxygen and nitrogen radicals or via Fas-Fas ligand interactions (Martinet and Kockx, 2001).

The global aim in the treatment of atherosclerosis is the prevention of cardiovascular complications (Libby, 2013). Lifestyle changes such as dietary lipid lowering, regular physical activity, smoke cessation, and weight control are necessary measures in the prevention of atherosclerosis. However, if lifestyle changes are not sufficient to modify risk factors of cardiovascular disease, treatment with lipid lowering drugs (e.g., statins, fibrates or PCSK9 inhibitors) is recommended. Given their high level of plasticity, macrophages represent an attractive alternative target for the development of anti-atherosclerosis therapies (Saha et al., 2009). Distinct anti-inflammatory strategies to treat or prevent atherosclerosis have recently emerged (Back and Hansson, 2015). Because cell death is a prominent feature of advanced atherosclerotic plaques with a major impact on atherogenesis and plaque destabilization (Kockx and Herman, 2000), this review will focus on the pharmacological modulation of macrophage death in atherosclerosis. As outlined in more detail below, plaque macrophages may undergo diverse types of death, ranging from standard mechanisms of death (apoptosis, necrosis) to less-well characterized mechanisms including necroptosis, pyroptosis, ferroptosis, parthanatos and autosis. Growing evidence indicates that pharmacological targeting of these types of death is a promising approach to stabilize vulnerable plaques and may contribute to the beneficial effects of currently applied plaque-stabilizing therapies (Martinet et al., 2011b, 2013; Karunakaran et al., 2016; Sergin et al., 2017).

## Targeting Macrophage Apoptosis

**FIGURE 1:**
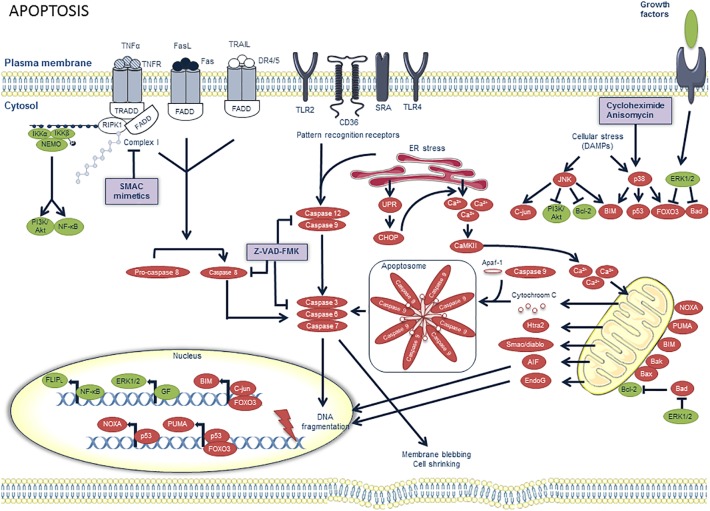
Overview of the apoptosis pathway and potential targets for pharmacological modulation. Pro-apoptotic factors are illustrated in red and prosurvival factors in green. Apoptosis can be initiated by multiple factors. Extrinsic pro-apoptotic pathways are activated when death ligands such as Fas ligand (FasL), TNFα and TNF-related apoptosis-inducing ligand (TRAIL) bind on death receptors (DR) such as Fas, TNFR1 and DR4/5, respectively. These receptors contain a death domain (DD) on the cytoplasmic side of the cell membrane, which can interact with other DD-containing proteins. The TNFα pathway is illustrated in more detail in Figure 3. Upon binding of FasL to Fas or TRAIL to DR4/5, Fas-associated death domain (FADD) is recruited. Consequently, FADD recruits pro-caspase 8 and converts it to active caspase 8. When caspase 8 is inhibited, for example by nitrosylation (due to high levels of NO production), or by peroxidation resulting from oxidative stress inside the plaque, a switch from apoptosis to necroptosis is observed, as further depicted in Figure 3. Other pro-apoptotic pathways include the activation of pattern recognition receptors (PRRs) by pathogen associated molecular patterns (PAMPs) and ER stress, both generating active caspases 9 and 12. Prolonged ER stress can activate the unfolded protein response (UPR), of which CCAAT-enhancer-binding protein homologous protein (CHOP) has been demonstrated to be a crucial pro-apoptotic effector in macrophages. CHOP promotes Ca^2+^ release from the ER, which in turn promotes activation of Ca^2+^/calmodulin-dependent protein kinase II (CamKII). The latter can induce Ca^2+^ uptake by the mitochondria which alters their membrane potential, thereby triggering the release of pro-apoptotic proteins, such as cytochrome C. Cytochrome C forms, together with caspase 9 and apaf-1, a complex called the apoptosome which also activates the effector caspases of apoptosis. Intrinsic pro-apoptotic pathways are activated upon exposure to ER stress, along with other cellular stressors or damage associated molecular patterns (DAMPs). They can activate mitogen-activated protein kinase (MAPK) signaling pathways which provide either pro-apoptotic (through JNK or p38 activation) or prosurvival signals (through ERK1/2 activation). The protein synthesis inhibitors anisomycin and cycloheximide are known to induce macrophage apoptosis through the p38 MAPK pathway. The pro-apoptotic pathways eventually lead to the activation of effector caspases 3, 6, and 7 which induce DNA fragmentation, membrane blebbing and cell shrinkage, all key features of apoptosis. Apoptosis can be pharmacologically inhibited by the pan-caspase inhibitor z-vad-fmk.

**FIGURE 2:**
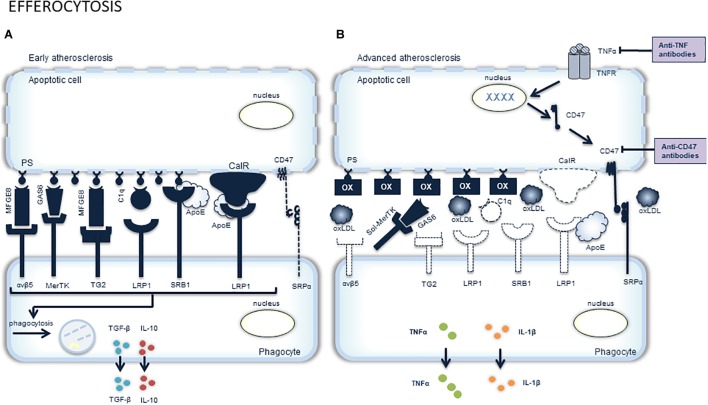
Overview of efferocytosis and potential targets for pharmacological stimulation. The clearance of apoptotic cells, a process called efferocytosis, is very important and closely regulated. Phagocytes responsible for efferocytosis have to discriminate viable, healthy cells from dying cells. This is mediated by the expression of “don’t eat me” molecules (e.g., CD47) on the surface of viable cells that interact with SIRPα (signal-regulatory protein α) on phagocytes and thereby prevent engulfment of the viable cell **(A)**. Dying cells on the other hand, express “eat me” molecules on their surface, such as calreticuline (calR) and phosphatidylserine (PS). These molecules interact with engulfment receptors on phagocytes (e.g., integrin αvβ5, Mer receptor tyrosine kinase (MerTK), transglutaminase 2 (TG2), low density lipoprotein receptor-related protein 1 (LRP1) and scavenger receptor B (SRB). The interaction between “eat me” molecules and engulfment receptors is mediated by bridging molecules [e.g., milk fat globule-EGF factor (MFGE), growth arrest-specific 6 (Gas6), and complement C1q]. In advanced atherosclerotic plaques, efferocytosis is impaired. This may be attributed to the altered phagocytic capacity of macrophages, VSMCs and dendritic cells. Moreover, dying cells in advanced plaques become poor substrates due to the decreased expression of “eat me” molecules and bridging molecules, as well as TNFα-induced expression of the “don’t eat me” molecule CD47 **(B)**. The latter can be inhibited by targeting either TNFα or CD47 with anti-TNFα or anti-CD47 antibodies, respectively. Another factor playing a role in impaired efferocytosis is the abundance of oxLDL in the plaque, which masks the “eat me” molecules and competes with the dying cells for macrophage engulfment. Competitive inhibition is also seen upon cleavage of the receptor MerTK, rendering an inactive, soluble form (sol-MerTK) that competes with the bridging molecules such as Gas6.

Macrophage apoptosis is an important feature of atherosclerotic plaque development and can be initiated by multiple factors such as oxidant stress, high concentrations of cytokines (e.g., TNFα), activation of the Fas death pathway by Fas ligand and endoplasmic reticulum (ER) stress (Seimon and Tabas, 2009) (Figure 1). In the past two decades, research directed at understanding the functional consequences of macrophage apoptosis in atherosclerosis revealed that the effect of macrophage death largely depends on the stage of the atherosclerotic plaque. Apoptosis of macrophages in early stages of the disease is considered beneficial as it limits lesion cellularity and suppresses plaque progression (Seimon and Tabas, 2009). Indeed, several experimental studies in mice suggest an inverse relationship between macrophage apoptosis and early lesion area. Reconstitution of ApoE^∗^3-Leiden mice with p53^-/-^ bone marrow, for example, resulted in reduced macrophage apoptosis, while macrophage content and lesion area increased significantly (van Vlijmen et al., 2001). Another study demonstrated reduced macrophage apoptosis and increased lesion size in LDLR^-/-^ mice reconstituted with bone marrow-derived hematopoetic cells lacking the pro-apoptotic protein Bax (Liu et al., 2005). Moreover, deletion of a macrophage survival protein, known as AIM (apoptosis inhibitor of macrophage) or IKKα, a protein directly associated with two major prosurvival pathways (PI3K/Akt and NF-κB), renders macrophages highly susceptible to oxLDL-induced cell death and reduces early atherosclerosis in LDLR^-/-^ mice (Arai et al., 2005; Babaev et al., 2016). Overall, these findings clearly illustrate that lesional macrophage apoptosis is necessary, at least initially, to reduce the pool of macrophages within the expanding plaques (Rayner, 2017). The results also imply that phagocytic clearance of apoptotic macrophages, better known as efferocytosis, is efficient in early lesions (Figure 2A). However, a large body of evidence indicates that efferocytosis in advanced lesions is impaired (Schrijvers et al., 2005) (Figure 2B). Several potential mechanisms of defective efferocytosis in atherosclerosis have been reported, but the most likely explanation is that efferocytosis itself becomes defective and/or that lesional apoptotic cells become poor substrates for efferocytosis (Yurdagul et al., 2018). Indeed, not only the expression and function of efferocytosis receptors (e.g., MerTK) and their bridging molecules (e.g., Gas6) are deficient in advanced plaques, dead cells in lesions also express lower amounts of “eat-me” signals (e.g., calreticulin), which prevents efficient clearance by phagocytic cells. Defective phagocytosis of apoptotic cells has a number of consequences that promotes the progression and complications of atherosclerotic plaques. Apoptotic cells that are not ingested, become secondarily necrotic, which can stimulate atherogenesis through induction of inflammation and enlargement of the necrotic core (Martinet et al., 2011a). Along these lines, it has been reported that apoptotic macrophages localize to sites of plaque rupture (Kolodgie et al., 2000), suggesting that macrophage death itself can promote plaque rupture. Non-cleared apoptotic cells are also an important source of tissue factor, which increases plaque thrombogenicity (Mallat et al., 1999). Accordingly, the impact of macrophage apoptosis in advanced plaques is far more complex as compared to early lesions.

Experimental evidence has demonstrated that inducing or enhancing the capacity of efferocytosis is a promising therapeutic path to promote the resolution of inflammation and to prevent formation of vulnerable plaques that are prone to rupture. One type of approach that may successfully target defective efferocytosis is the use of antibodies that block the anti-phagocytic ‘don’t eat me signal’ CD47. TNFα promotes CD47 overexpression in atherosclerotic plaques via NF-κB and renders vascular cells resistant to phagocytic clearance (Kojima et al., 2016). Atherosclerotic mice treated with anti-CD47 antibodies display less apoptotic debris and develop significantly smaller atherosclerotic plaques as compared to mice treated with control antibodies (Kojima et al., 2016). Of note, concomitant inhibition of CD47 and TNFα using anti-CD47 antibody therapy and commercially available anti-TNFα antibodies such as infliximab or etanercept offers a synergistic benefit in the clearance of apoptotic cells (Kojima et al., 2016). Given that the risk of future cardiovascular events can be reduced in patients with rheumatoid arthritis undergoing anti-TNFα therapy, there is a strong rationale for combining anti-inflammatory and pro-efferocytic therapies for the treatment of advanced atherosclerosis (Kojima et al., 2017). However, anti-CD47 antibodies also promote erythrophagocytosis so that dose optimization and changes in treatment regimen may be required to avoid anemia. Another type of approach is to enhance the expression of efferocytosis-related genes. Glucocorticoids, the most widely used anti-inflammatory drugs, are known to enhance both short-term and continued efferocytosis, at least *in vitro*. Short-term phagocytosis of apoptotic cells is enhanced mainly by upregulation of the MerTK receptor and bridging molecule C1q expression levels, while continued phagocytosis is promoted via the induction of lipid sensing nuclear receptors (e.g., LXR, PPARδ) and uncoupling protein 2 (UCP2) (Garabuczi et al., 2015). However, glucocorticoids can also enhance efferocytosis by stimulating the release of annexin A1 (Maderna et al., 2005; Scannell et al., 2007), which is highly expressed in resting macrophages. Once secreted, annexin A1 binds to phosphatidylserine (PS) at the surface of apoptotic cells, and thereby serves as a bridging molecule between PS and the annexin receptor on the macrophage surface. Recently, de Jong et al. (2017) demonstrated that annexin A1 levels in the vessel wall and circulating plasma negatively correlate with neointima size, which confirms its reparative role by enhancing efferocytosis. However, the long-term use of corticosteroids is associated with many undesirable and potentially lethal side effects. It may lead to vascular injury and can stimulate experimental atherosclerosis so that prolonged therapy with this type of medication should be avoided whenever possible. Yet another exciting approach to clear apoptotic cells in advanced plaques would be to increase the production of long-chain fatty acid-derived lipid mediators, called specialized proresolving mediators (SPMs), including resolvin D1 (RvD1), RvD2, RvE1, and RvE2. Resolvins mediate resolution by blocking the production of pro-inflammatory mediators (i.e., chemokines and cytokines) and leukocyte trafficking to sites of inflammation (Fredman and Serhan, 2011), albeit they also promote efferocytosis through p50/p50-homodimer-mediated repression of TNFα production (Lee et al., 2013). Interestingly, levels of SPMs (in particular RvD1) in vulnerable regions of atherosclerotic plaques are significantly decreased (Fredman et al., 2016), possibly through cleavage of MerTK, as mice with cleavage-resistant MerTK have increased circulating levels of SPMs (Cai et al., 2016). Administration of RvD1 to LDLR^-/-^ mice during plaque progression restores RvD1 levels to that of less advanced lesions and improves plaque stability by enhancing lesional efferocytosis (Fredman et al., 2016). Also delivery of other SPMs (e.g., RvD2, Maresin-1, RvE1) favors a proresolving milieu and prevents atheroprogression in mice (Hasturk et al., 2015; Viola et al., 2016). Finally, it should be mentioned that pharmacological inhibition of macrophage apoptosis obviously may help to inhibit the accumulation of non-phagocytized apoptotic debris and plaque development. A recent example is the study of Tian et al. (2017) showing that the apoA-I mimetic peptide D4F protects macrophages from oxLDL-induced apoptosis by suppressing the activation of the Fas/FasL pathway, which reduces plaque formation in atherosclerotic ApoE^-/-^ mice.

Based on the abovementioned findings, we may conclude that induction of macrophage apoptosis in plaques with impaired efferocytosis represents a certain risk. However, macrophages that accumulate in the plaque actively promote degradation of the extracellular matrix and contribute to death of VSMCs. From this perspective, selective removal of macrophages via macrophage-specific initiation of cell death may also have plaque-stabilizing effects. To further clarify this issue, Gautier et al. (2009) developed CD11c-diptheria toxin (DT) receptor (DTR) transgenic mice that show macrophage apoptosis after administration of DT. Sustained macrophage apoptosis was associated with increased plaque inflammation and accelerated plaque progression. These results are different from those of Stoneman et al. (2007), who reported that acute induction of macrophage apoptosis in a similar animal model (CD11b-DTR mice) had no effect on plaque extent or composition. The difference in the final outcome of both studies could be related to the use of two different mouse models (CD11b-DTR versus CD11c-DTR). Because monocytes are CD11b^high^ cells, there is a possibility that they are (more) extensively depleted in CD11b-DTR mice (50% reduction in circulating monocytes) as compared to CD11c-DTR mice so that their recruitment to the lesion and the impact on vascular inflammation and plaque development is significantly impaired. On the other hand, DT-treated CD11c-DTR mice revealed an elevation in plasma cholesterol, which may have contributed to an increase in lesion size (Gautier et al., 2009). In addition to the use of genetically modified mice, our laboratory developed different pharmacological strategies to selectively deplete macrophages in atherosclerotic plaques through apoptosis induction. Plaque macrophages are metabolically highly active, thus also more sensitive to protein synthesis inhibitors as compared to other cell types in the vessel wall including VSMCs and endothelial cells. As a consequence, local administration of the protein synthesis inhibitor cycloheximide to rabbit plaques depletes the macrophage content via p38 MAPK-mediated apoptosis induction without altering VSMC viability or other obvious detrimental effects (Croons et al., 2007). The protein synthesis inhibitor anisomycin provides similar results, with a mechanism of action analogous to cycloheximide (Croons et al., 2009). Other strategies that allow the selective removal of plaque macrophages via apoptosis include the induction of ER stress by exogenous NO donors (De Meyer et al., 2003; Martinet et al., 2007a), or the inhibition of inositol monophosphatase by lithium chloride (De Meyer et al., 2011). Although these strategies are successful in a preclinical setting and look promising, there are several drawbacks that may hamper clinical applications (Croons et al., 2010; Martinet et al., 2011b). First, depletion of peripheral blood monocytes should be avoided, because they play a critical role in both innate and adaptive immunity. Local delivery of macrophage depleting drugs (e.g., with drug-eluting stents or targeted nanoparticles) can decrease or avoid unwanted systemic effects. Second, additional pro-efferocytic therapy will be required to prevent accumulation of free (non-engulfed) apoptotic cells. Because the phagocytic capacity of plaques will be compromised by depleting macrophages, this prerequisite may be hard to accomplish. In addition, combined treatment (for example with lipid lowering drugs such as statins) may be needed to counteract re-infiltration of circulating monocytes after macrophage depletion. Finally, it would be recommendable to conceive therapeutic strategies that selectively promote apoptosis in early lesional macrophages and/or that selectively prevent cell death in advanced lesions, which is again not self-evident.

## Targeting Macrophage Necrosis

### Passive Necrosis

Necrotic cell death is characterized by an increased cell volume (oncosis), organelle swelling and chromatin condensation, which eventually culminates in plasma membrane rupture and the release of intracellular compounds (Majno and Joris, 1995). Macrophage necrosis is a key feature in the pathogenesis of atherosclerosis (Crisby et al., 1997; Tabas, 2005), and triggers the formation and enlargement of a central necrotic core, which plays a pivotal role in unstable atherosclerotic plaques (Tabas, 2005). Indeed, 80% of necrotic cores in advanced human atherosclerotic plaques are larger than 1 mm^2^, which compromises > 10% of the lesion area (Virmani et al., 2007). However, in 65% of plaque ruptures, the necrotic core occupies > 25% of the plaque area (Virmani et al., 2007). Due to a lack of reliable methods to detect and quantify necrosis in tissue, plaque necrosis has not been extensively studied. Plaque necrosis can be triggered by multiple factors including high levels of oxidative stress, an overload of intracellular Ca^2+^ and cellular ATP depletion. Moreover, as mentioned above, impaired efferocytosis in advanced plaques causes accumulation of apoptotic bodies, which undergo secondary necrosis (Martinet et al., 2011a). Necrotic macrophages do not only contribute to the formation and enlargement of the necrotic core, they are also a source of pro-inflammatory cytokines and damage associated molecular patterns (DAMPs) (Seimon and Tabas, 2009). The release of DAMPs promotes inflammation in the plaque, thereby causing plaque instability. High mobility group box 1 protein (HMGB1) is one of the best studied DAMPs in atherosclerosis. Macrophages in atherosclerotic plaques are a major source of HMGB1 production (Kalinina et al., 2004). Once released in the extracellular space, HMGB1 interacts with different receptors including receptor for advanced glycation end-products (RAGE). Binding of HMGB1 triggers the transcription of pro-inflammatory cytokines in a NF-κB dependent manner, thereby promoting further plaque development (de Souza et al., 2012). Experimental evidence has shown that HMGB1 expression increases during atherogenesis (Kalinina et al., 2004). Consistently, a more recent study has demonstrated that HMGB1 is highly expressed in mouse atherosclerotic plaques. Neutralization of HMGB1 in ApoE^-/-^ mice reduces plaque area through inhibition of immune cell accumulation and macrophage migration (Kanellakis et al., 2011). Interestingly, mounting experimental evidence suggests that statins attenuate plaque formation partly by reducing HMGB1 and RAGE expression (Cuccurullo et al., 2006; Calkin et al., 2008; Yin et al., 2010). Moreover, administration of simvastatin to ApoE^-/-^ mice reduces vascular inflammation via downregulation of the HMGB1-RAGE axis (Liu et al., 2013). Besides inhibiting DAMPs, directly targeting necrotic cell death by using antioxidant therapy could be an alternative strategy for plaque stabilization. Several epidemiological, clinical, and experimental studies have been conducted to evaluate possible beneficial effects of antioxidant vitamins such as vitamins C and E on atherogenesis. However, there is no convincing evidence that anti-oxidant therapy attenuates atherosclerotic plaque progression (Cherubini et al., 2005). Targeting specifically mitochondrial ROS by administration of NecroX-7, which acts as a scavenger of mitochondrial ROS and peroxynitrite, could be an alternative approach to inhibit necrotic cell death (Kim et al., 2010). Recently, our laboratory demonstrated that NecroX-7 treatment reduces the necrotic area without affecting plaque size in ApoE^-/-^ mice (Grootaert et al., 2016). Moreover, NecroX-7 improves important features of plaque stability such as lowering plaque inflammation, reducing oxidative stress and increasing collagen content and fibrous cap thickness (Grootaert et al., 2016). Currently, a phase 2 clinical study is evaluating the efficacy, safety and pharmacokinetics of a single intravenous injection of NecroX-7 before percutaneous coronary intervention in patients with ST-segment elevated myocardial infarction (STEMI) (^1^identifier NCT02770664). As atherosclerosis is a chronic disease, the evaluation of chronic administration of NecroX-7 should be included in future clinical trials.

### Necroptosis

**FIGURE 3:**
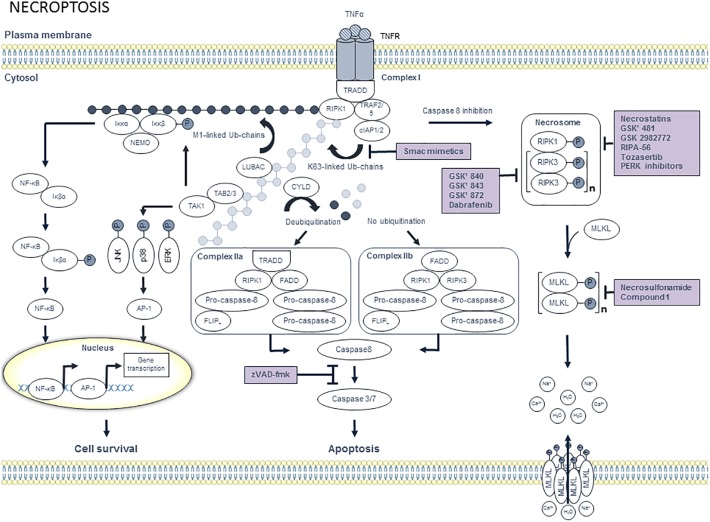
Overview of the necroptosis pathway and potential targets for pharmacological inhibition. Binding of tumor necrosis factor α (TNFα) to trimeric tumor necrosis factor receptor 1 (TNFR1) leads to the recruitment of tumor TNFR1-associated death domain (TRADD) and receptor interacting protein kinase 1 (RIPK1). Subsequently, TNFR-associated factor 2 (TRAF2), TRAF5, cellular inhibitor of apoptosis protein 1 (cIAP1) and cIAP2 are recruited to TRADD, thereby forming complex I. CIAP1/2 then ubiquitinates RIPK1 with K63-linked ubiquitin chains, which allows the recruitment of linear ubiquitin chain assembly complexes (LUBAC). Subsequently, M1-linked ubiquitin chains are generated by LUBAC and added to RIPK1. Both M1- and K63-ubiquitin chains serve as a scaffold for the recruitment of IKKα/IKKβ/NEMO and TAB2/TAB3/TAK1, respectively. Subsequently, TAK1 phosphorylates IKKβ as well as the downstream MAPKs including JNK, p38 and ERK, which activate the transcription factor AP-1. Phosphorylated IKKβ activates IκBα, which results in the release of nuclear factor κB (NF-κB). Finally, NF-κB translocates toward the nucleus, where it induces transcriptional upregulation of pro-survival genes. Cell survival can be inhibited by targeting cIAP1/2 with smac mimetics. When RIPK1 is deubiquitinated by cylindromatosis protein (CYLD), RIPK1 dissociates from complex I and engages with FADD, which in turn recruits pro-caspase 8 and FLICE like protein long isoform (FLIP_L_) heterodimer and pro-caspase 8 homodimer to form complex IIa. Pro-caspase 8 and FLIP_L_ heterodimer inhibits activation of caspase 8, thereby promoting cell survival. In contrast, pro-caspase 8 homodimer generates active caspase 8, which initiates caspases 3 and 7 to induce apoptotic cell death. When RIPK1 is not ubiquitinated, complex IIb will be formed. Complex IIb consists of RIPK1, RIPK3, pro-caspase 8 and FLIP_L_ and is also known as the ripoptosome. In contrast to complex IIa, the kinase activity of RIPK1 is crucial to induce apoptosis via complex IIb. Apoptotic cell death can be pharmacologically inhibited by pan-caspase inhibitors such as zVAD-fmk. When RIPK1 deubiquitination is inactivated and caspase 8 is inhibited, RIPK1 will dissociate from complex I. In the cytosol, RIPK1 binds to RIPK3 and subsequently a series of auto- and trans-phosphorylations of RIPK1 and RIPK3 occurs. Phosphorylated RIPK3 then recruits and phosphorylates mixed lineage kinase domain-like protein (MLKL), thereby promoting MLKL oligomerization. Next, MLKL oligomers migrate to the plasma membrane and execute necroptosis by the formation of pores and by the deregulation of sodium and calcium channels. Pharmacological suppression of necroptosis may occur by compounds that inhibit RIPK1, RIPK3, or MLKL.

For many years, necrosis has been considered as an uncontrolled way for a cell to die. However, research in the field of cell death drastically changed by the discovery of small molecules, termed necrostatins, which inhibit receptor-interacting protein kinase (RIPK)1 to induce necroptosis in TNFα-treated cells (Degterev et al., 2005, 2008) (Figure 3). This discovery led to the characterization of downstream necroptosis mediators, namely RIPK3 and mixed lineage kinase domain-like protein (MLKL) (Cho et al., 2009; He et al., 2009; Zhang et al., 2009). In addition to TNFα, other necroptosis triggers have been identified including TNF-related apoptosis inducing ligand (TRAIL), first apoptotic signal ligand (FasL), interferons, toll-like receptor ligands, and virus-activated pathways (Vanden Berghe et al., 2014). Nonetheless, TNFα-induced necroptosis is currently the best-characterized necroptosis pathway. The response of cells to TNFα is complex. In most cases, binding of TNFα leads to ubiquitination of RIPK1 by cellular inhibitors of apoptosis (cIAP1/2) and linear ubiquitin chain assembly complexes (LUBAC), followed by the activation of cell survival pathways including NF-κB and MAPK pathway. However, de-ubiquitination of RIPK1 promotes cell death signaling pathways, such as caspase-dependent apoptosis and RIPK3-MLKL-mediated necroptosis (Dondelinger et al., 2016). Necroptosis and apoptosis pathways seem to compete with each other. When caspase 8 is inhibited by pharmacological or physiological stimuli, the necroptosis proteins RIPK1 and RIPK3 will be phosphorylated (Declercq et al., 2009). Subsequently, RIPK3 induces phosphorylation of MLKL, which triggers MLKL oligomerization. Finally, MLKL oligomers associate with the plasma membrane, which causes plasma membrane disruption and the release of DAMPs (Dondelinger et al., 2014; Wang et al., 2014). Recently, it has been demonstrated that gene expression of the necroptosis mediators RIPK3 and MLKL is elevated in human atherosclerotic plaques. Moreover, RIPK3 and MLKL mRNA are specifically upregulated in subjects with unstable compared to stable atherosclerotic plaques (Karunakaran et al., 2016). This finding is supported by another study demonstrating that the protein levels of RIPK1 and RIPK3 are increased in advanced human atherosclerotic lesions (Tian et al., 2016). In addition, RIPK3 expression is elevated in advanced plaques of LDLR^-/-^ mice. Interestingly, RIPK3 gene expression in macrophages increases during the development of atherosclerosis, which suggests that macrophage necroptosis plays a role in advanced plaques. Indeed, RIPK3 deficiency in bone-marrow-derived cells reduces advanced atherosclerotic lesions in ApoE^-/-^ and LDLR^-/-^ mice (Lin et al., 2013). Overall, these findings clearly illustrate a role for RIPK3-mediated macrophage necroptosis in atherosclerosis. Besides these genetic studies, Karunakaran et al. (2016) demonstrated that pharmacological inhibition of RIPK1 by Nec-1s reduces plaque size and promotes plaque stability in ApoE^-/-^ mice with established atherosclerotic lesions. Additional *in vitro* experiments have unraveled the underlying mechanism by which necroptosis is induced in atherosclerosis. During plaque development, oxidized LDL increases ROS-mediated RIPK3 and MLKL gene expression in macrophages, which leads to necroptosis (Karunakaran et al., 2016). Overall, these data demonstrate that inhibition of macrophage necroptosis could be a promising therapeutic strategy to prevent the development of a vulnerable plaque. During the past decade, several research groups have been focusing on the development of RIPK1 inhibitors. As a result, a phase 2 clinical study is ongoing to evaluate the effect of the RIPK1 inhibitor GSK2982772 on psoriasis, ulcerative colitis and rheumatoid arthritis (^1^identifier NCT 02776033, NCT02903966, and NCT 02858492). However, potential beneficial effects of GSK2982772 on atherosclerosis have not been studied yet.

### Pyroptosis

**FIGURE 4:**
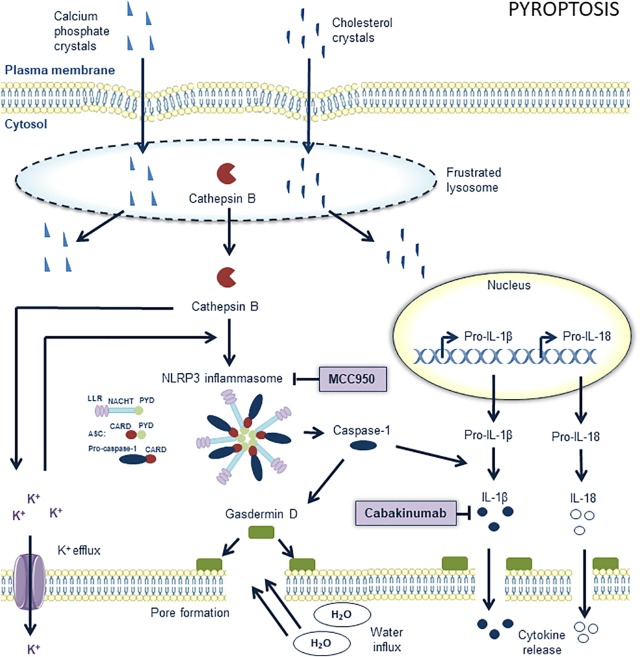
Overview of the pyroptosis pathway and potential targets for pharmacological inhibition. The nucleotide-binding oligomerization domain-like receptor (NLR) family, pyrin domain containing 3 (NLRP3) inflammasome is a key component in pyroptosis. It contains procaspase 1 and processes it into active caspase 1, the main effector of pyroptosis. MCC950 is a potent and selective inhibitor of the NLRP3 inflammasome in macrophages in atherosclerotic lesions. The upstream pathways of the NLRP3 inflammasome are not completely elucidated but cholesterol crystals and calcium phosphate crystals are known to play a role. They “frustrate” lysosomes which subsequently lyse and release their content into the cytoplasm. The subsequent release of cathepsin B out of these lysosomes into the cytoplasm promotes potassium efflux. These events promote the assembly of the NLRP3 inflammasome which consists of conserved domains such as C-terminal leucine-rich repeats (LRRs), a central nucleotide domain, called NACHT, and an N-terminal pyrin domain (PYD). Assembly of the inflammasome occurs through the interactions of pyrin domains (PYD) and caspase recruitment domains (CARD). The apoptosis-related speck-like protein containing CARD (ASC) is composed of a PYD and CARD domain and may serve as a link between LRR-NACHT-PYD and CARD bound to procaspase 1. Active caspase 1 cleaves gasdermin D to induce pore formation and processes pro-IL-18 and pro-IL-1β into active interleukins, which are released out of the cells through the formed pores. IL-1β can be pharmacologically targeted with anti-IL-1β antibodies such as canakinumab. Further pore formation by gasdermin D eventually leads to cell swelling and osmotic lysis.

Macrophages may undergo other types of regulated necrosis, even though their significance in atherosclerosis is not always clear-cut. Among the most well-defined is pyroptosis, a pro-inflammatory form of regulated cell death that is triggered by the activation of caspase-1 (Xu et al., 2018) (Figure 4). Activation of this inflammatory protease requires a large supramolecular complex, known as an inflammasome. The NLR protein-3 (NLRP3) inflammasome is currently the best characterized inflammasome and consists of NLRP3, ASC, and procaspase-1. Mounting evidence suggests that oxLDL as well as crystals of cholesterol and calcium phosphate in atherosclerotic plaques activate NLRP3 inflammasomes through lysosomal rupture and subsequent cathepsin release (Karasawa and Takahashi, 2017; Grebe et al., 2018), which in turn leads to cleavage and activation of procaspase-1. The activated caspase-1 exerts proinflammatory effects by converting pro-IL-1β and pro-IL-18 into their bioactive form. Activated caspase-1 also leads to processing of gasdermin D and the rapid formation of plasma membrane pores, which in turn allows water influx, cell swelling and osmotic lysis. Interestingly, secretion of IL-1β and IL-18 does not require lysis and is temporally associated with gasdermin D-dependent pore formation, suggesting that these pores are sufficient to mediate cytokine release. Recent studies have shown that components of the NLRP3 inflammasome in human plaques are mainly expressed in macrophages (Shi et al., 2015), and that pyroptosis in plaque macrophages may promote necrotic core formation and plaque instability in advanced lesions (Xu et al., 2018). Because both NLRP3 and caspase-1 deficiency decreases atherosclerosis in ApoE^-/-^ mice (Gage et al., 2012; Usui et al., 2012; Zheng et al., 2014), therapeutic modulation of NLRP3 or caspase-1 activity is likely to offer significant health benefits for patients with severe atherosclerosis. This assumption is further supported by several epidemiological studies showing that patients with coronary atherosclerosis display high aortic expression of NLRP3, which is directly correlated to disease severity and clinical risk factors for cardiovascular disease (e.g., hypertension, diabetes, smoking, LDL cholesterol) (Zheng et al., 2013). Others reported increased expression of NLRP3, caspase-1, IL-1β, and IL-18 in carotid plaques as compared to non-atherosclerotic arteries with the highest expression levels in unstable lesions (Shi et al., 2015). Besides modulating NLRP3 or caspase-1 activity, direct targeting of IL-1β in atherosclerosis, without affecting cholesterol levels, is an interesting alternative approach that was recently evaluated in the CANTOS trial using canakinumab, a human monoclonal antibody that binds to IL-1β and thereby blocks the interaction of IL-1β with its receptor and subsequent downstream pro-inflammatory signaling events. CANTOS clearly demonstrated that statin-treated patients with residual inflammatory risk, as determined by elevated CRP levels, benefit from additional anti-inflammatory therapy (Ridker et al., 2017), which is in line with previous findings in mice showing that monoclonal antibodies targeting IL-1β inhibit atherosclerotic plaque formation (Bhaskar et al., 2011). However, according to a more recent study, IL-1β neutralization in advanced lesions of ApoE^-/-^ mice induces loss of VSMC and collagen within the fibrous cap, whereas the macrophage content increased (Gomez et al., 2018). This finding indicates that IL-1β has an unexpected atheroprotective role in late-stage atherosclerosis, most likely by stimulating cell proliferation (Libby et al., 1988). Accordingly, it will be critical to determine which patients will benefit most from anti-IL-1β therapy, and more importantly, which patients might develop adverse effects (Gomez et al., 2018).

### Ferroptosis

**FIGURE 5:**
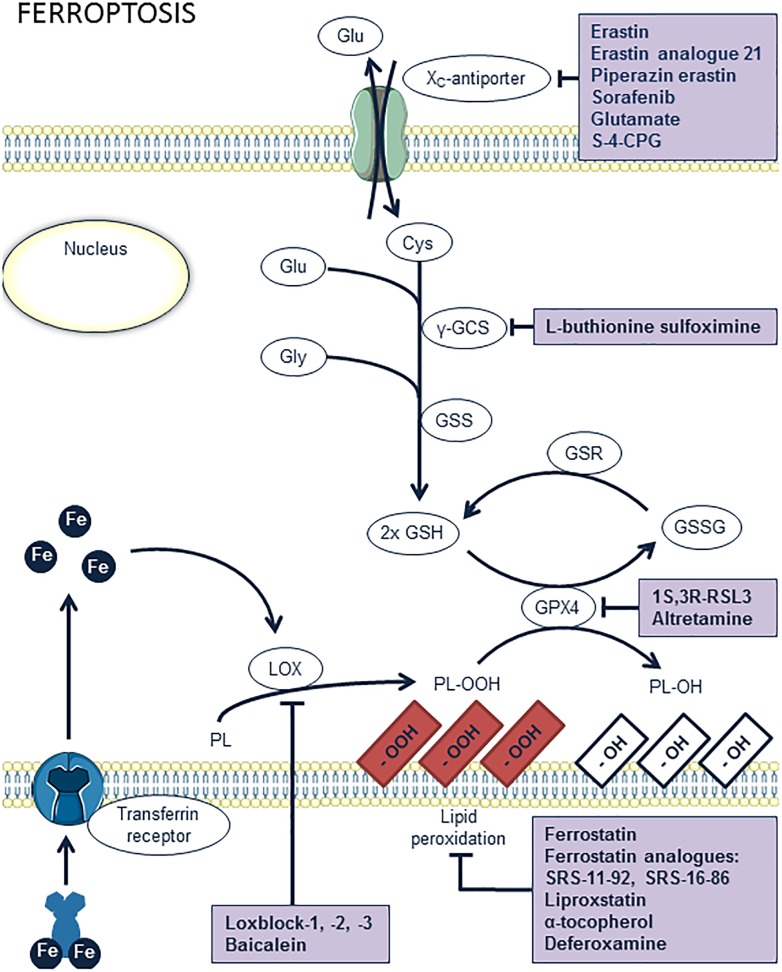
Overview of the ferroptosis pathway and potential targets for pharmacological inhibition. Lipoxygenases (LOX) and iron catalyze the peroxidation of phospholipids (PL). Peroxidized lipids are reduced by glutathion peroxidase 4 (GPX4), upon simultaneous oxidation of glutathion (GSH). However, excessive lipid peroxidation of membrane phospholipids induces ferroptosis. This may occur when GPX4 is inhibited, e.g., by 1S,3R-RSL3 or altretamin, or when glutathion is absent. The latter can result from inhibition of glutathion synthesis by L-buthionine sulfoximine or from a lack of cystein inside the cells through inhibition of the x_c_-antiporter system, e.g., by erastin or analogs, sorafenib, glutamate, or *S*-4-carboxyphenylglycine (*S*-4-CPG). Ferrostatin, liproxstatin, vitamin E, and deferoxamine are antioxidant compounds known to inhibit ferroptosis. Another way of inhibiting ferroptosis is provided by inhibition of lipid peroxidation itself by blocking LOX enzymes with loxblock-1, -2, -3, or baicalein.

Ferroptosis is a form of regulated necrosis that is associated with iron-dependent accumulation of lipid hydroperoxides (Dixon et al., 2012; Stockwell et al., 2017) (Figure 5). This iron-catalyzed form of necrosis can be induced by erastin (Cao and Dixon, 2016), which inhibits the membrane-bound cystine/glutamate antiporter $X_{c}^{-}$. Blockage of this antiporter impairs the cellular uptake of cystine, an essential precursor in the synthesis of the cellular antioxidant glutathione (GSH). This intracellular deficit of GSH triggers the accumulation of reactive oxygen species (ROS), which causes cells to die by excessive oxidation of the membrane lipids (Cao and Dixon, 2016). To the best of our knowledge, ferroptosis has not yet been investigated in atherosclerosis, but is gaining considerable attention. Indeed, given that lipid peroxidation, intraplaque hemorrhages and iron deposition are hallmarks of advanced human plaques, which is indirect evidence for the initiation of ferroptosis, we assume that macrophage ferroptosis has a major role in atherosclerotic plaque destabilization. Glutathione peroxidase 4 (GPX4) can be considered to be one of the most important anti-oxidant enzymes in mammals because of its unique activity to reduce phospholipid hydroperoxides. A study by Seiler et al. (2008) underlined the importance of GPX4 in the prevention of ferroptosis by showing that inducible GPX4 gene inactivation in mice or cultured cells caused significant cell death due to excessive lipid peroxidation, which is a main feature of ferroptosis. Conversely, overexpression of GPX4 removes oxidative lipid modifications and inhibits plaque development in ApoE^-/-^ mice (Guo et al., 2008), thereby confirming the possible role of ferroptosis in cardiovascular disease. Ferrostatin (Fer-1) is the first pharmacological compound that was identified as a selective and potent inhibitor of ferroptosis in *in vitro* experiments (Dixon et al., 2012). It is thought to exert its anti-ferroptosis activity by preventing oxidative damage to membrane lipids. Unfortunately, Fer-1 suffers from inherent stability problems (hydrolysis of ester moiety), which drastically limits the application of these molecules *in vivo*. Recently, Fer-1 analogs with improved potency and ADME properties have been designed (Hofmans et al., 2016). Analogous with other anti-ferroptosis drugs (e.g., liproxstatins, anti-oxidants such as vitamin E), these molecules are able to prevent ferroptotic cell death, though further investigation is needed to determine whether these compounds are able to inhibit atherosclerosis.

### Parthanatos

**FIGURE 6:**
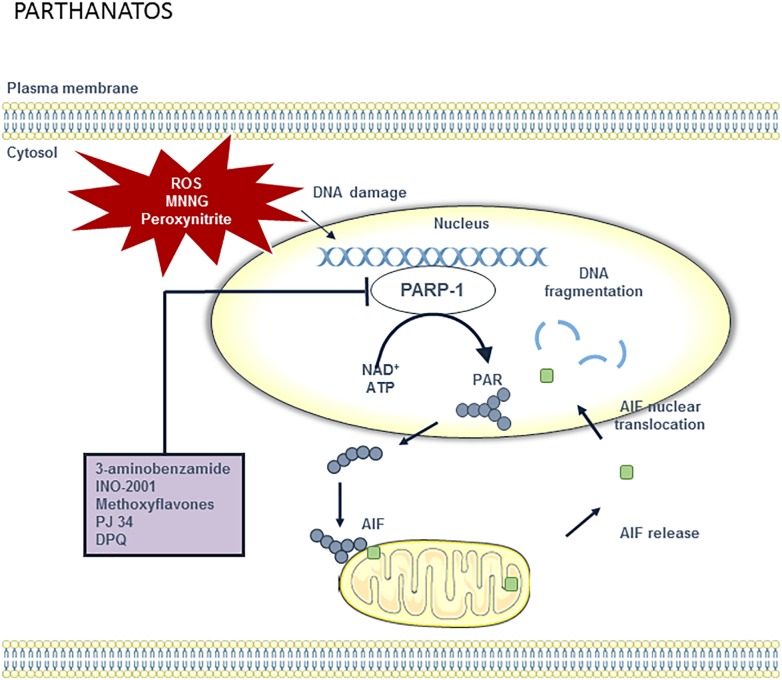
Overview of the parthanatos pathway and potential targets for pharmacological inhibition. DNA damage can be caused by different stimuli including ROS, methylnitronitrosguanidine (MNNG) and peroxynitrite. When DNA is damaged, PARP-1 activity will increase in order to repair the DNA. However, poly (ADP-ribose) polymerase 1 (PARP-1) overactivation results in the depletion of the cellular pool of nicotinamide adenine dinucleotide (NAD^+^) and ATP, which are used to synthesize poly ADP-ribose (PAR) polymer. PAR polymers migrate to the mitochondria, where they directly bind to apoptosis-inducing factor (AIF). AIF is then released from the mitochondria and translocated to the nucleus. Once in the nucleus, AIF causes large-scale DNA fragmentation and chromatin condensation. Parthanatos can be blocked by PARP-1 inhibitors such as 3-aminobenzamide, INO-1001, methoxyflavones, PJ34, and DPQ.

Another emerging type of regulated leukocyte necrosis in cardiovascular disease is parthanatos (Barany et al., 2017), a form of cell death that is different from the other cell death processes described above (Figure 6). Parthanatos is driven by hyperactivation of poly(ADP-ribose) polymerase-1 (PARP-1) and occurs in response to oxidative damage of cellular DNA. By forming poly(ADP-ribose) (PAR) polymers, PARP-1 overactivation depletes the cellular pool of nicotinamide adenine dinucleotide (NAD^+^) and ATP, yet this does not seem to be the primary cause of cell death (Fatokun et al., 2014). One of the key processes of parthanatos is the binding of poly(ADP-ribose) polymers to apoptosis-inducing factor (AIF), which promotes the release of AIF into the cytosol and its translocation into the nucleus, where it mediates large-scale DNA fragmentation and chromatin condensation. Pharmacological inhibitors of this process can efficiently delay parthanatos in multiple cell types. Among the different therapeutic opportunities in the parthanatos cascade (e.g., inhibition of PARP, PAR, AIF release, or nuclear translocation), only PARP inhibitors are the most advanced in development (Fatokun et al., 2014). Currently, parthanatos is a poorly studied phenomenon in atherosclerosis, though several lines of evidence indicate that parthanatos in plaque macrophages could play a major role in atherogenesis and represents an interesting drug target. First, advanced plaques reveal high levels of oxidative stress and tissue damage through formation of peroxynitrite (Martinet and Kockx, 2001). Once formed, peroxynitrite can oxidize a variety of biomolecules including DNA. Elevated levels of the oxidative DNA damage marker 7,8-dihydro-8-oxoguanine and an increased number of DNA strand breaks have been reported in both human and experimental plaques (Martinet et al., 2001, 2002). Most interestingly, oxidative DNA damage was associated with the upregulation of PARP-1 (and other DNA repair enzymes), predominantly in the macrophage-derived foam-cells of the plaque. Second, pharmacological inhibition of PARP-1 in atherosclerosis attenuates plaque development in ApoE^-/-^ mice. Moreover, a reduction of PARP-1 activity enhances plaque stability and promotes the regression of pre-established plaques. Inadequate formation of plaques after PARP-1 inhibition may result from impaired translocation of NF-κB into the nucleus, followed by a reduction in inflammatory mediators (e.g., VCAM-1, MCP-1) and monocyte recruitment (Oumouna-Benachour et al., 2007; Xie et al., 2009). PARP-1 inhibition may also have a major impact on endothelial function, foam cell formation, lipid metabolism and the induction of cell death (switch from necrosis to apoptosis), all of which are central to the pathogenesis of atherosclerosis (Xu et al., 2014).

## Targeting Macrophage Autophagy

**FIGURE 7:**
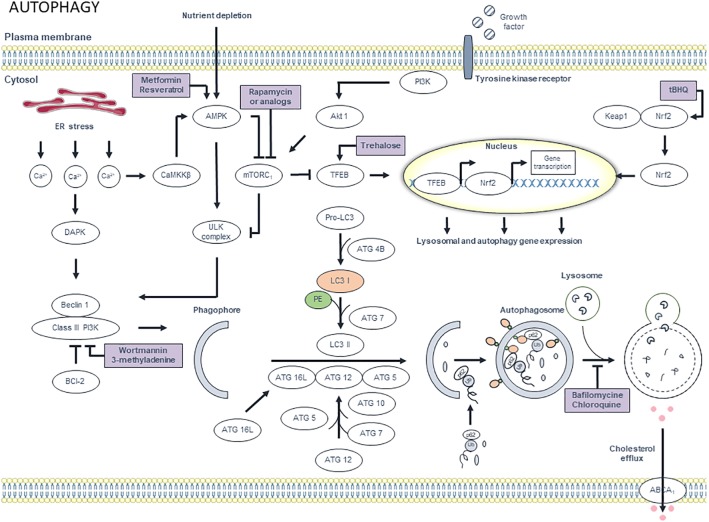
Overview of the autophagy pathway and potential targets for pharmacological modulation. Different extracellular signals such as amino acids, growth factors and insulin, activate mammalian target of rapamycin complex 1 (mTORC_1_) via PI3K and Akt1 signaling, thereby inhibiting autophagy. However, upon deprivation of nutrients and growth factors, AMP-dependent protein kinase (AMPK) is activated. AMPK inhibits mTORC_1_, which results in the activation of ULK complex. Interestingly, its activation can also occur independently from mTORC_1_. ULK complex, in turn, activates class III PI3K via phosphorylation of beclin 1. Subsequently, class III PI3K generates phosphatidylinositol 3-phosphates (PI3P), which are essential for the synthesis of the phagophore membrane. Further membrane elongation involves two ubiquitin-like conjugation systems. In the first system, ATG12 is conjugated to ATG5 via a reaction that involves ATG7 and ATG10. Subsequently, ATG12-ATG5 complex binds to ATG16L. ATG12-ATG5-ATG16L associates with the phagophore. The second system involves the conjugation of LC3 to phosphatidylethanolamine (PE). First, pro-LC3 is processed by Atg4B, which results in cytosolic LC3-I. ATG7 then adds phosphatidylethanolamine (PE) to LC3-I to form LC3-II, which associates with the phagophore membrane. Misfolded proteins and aggregates are tagged with ubiquitin chains, which are a substrate for p62. Protein – p62 complexes aggregate and migrate to the autophagosomal membrane, where p62 interacts with LC3. After the formation of the autophagosome, the outer membrane of the phagosome fuses with a lysosome, thereby forming an autolysosome. Finally, the internal material is degraded by lysosomal enzymes and nutrients and metabolites are recycled. Efflux of cholesterol via ABCA_1_ transporters is promoted by autophagy. Interestingly, autophagy can be regulated at the transcriptional level. mTORC_1_ inactivation results in nuclear translocation of transcription factor EB (TFEB). Once in the nucleus, TFEB triggers the transcription of autophagy and lysosomal genes. Moreover, oxidant stress can trigger autophagy by disrupting the interaction between nuclear factor erythroid-derived 2-like 2 (Nrf2) and Kelch-like ECH-associated protein 1 (Keap1). Nrf2 then translocates to the nucleus where it upregulates the transcription of different autophagy-related genes including p62. Additionally, calcium homeostasis plays a role in the autophagy pathway. ER stress causes calcium release, which activates proteases such as death-associated protein kinase (DAPK) and Ca^2+^/calmodulin-dependent protein kinase kinase β (CaMKKβ). The latter induces autophagy via inhibition of mTORC_1_ in an AMPK-dependent manner. DAPK induces autophagy by phosphorylating beclin 1 and thereby activating class III PI3K. Autophagy can be stimulated pharmacologically by targeting AMPK using metformin and resveratrol. Rapamycin (or analogs) induce autophagy via mTORC_1_ inhibition. Induction of transcription of lysosomal and autophagy genes by targeting Nrf2 and TFEB with tert-butylhydroquinone (tBHQ) and trehalose, respectively, is an alternative approach for autophagy induction. Autophagy can be inhibited by targeting class III PI3K with wortmannin and 3-methyladenine. Alternatively, the autophagic process can be blocked by targeting lysosomes with bafilomycin and chloroquine.

Autophagy is an evolutionary conserved physiological process in the body that maintains intracellular homeostasis by sequestrating unnecessary or dysfunctional cellular components in double membrane structures, called autophagosomes (Dikic and Elazar, 2018) (Figure 7). The latter fuse with lysosomes, where the contents are degraded by acid hydrolases. As the autophagic process allows recycling of macromolecules and energy, basal autophagy primarily has cytoprotective functions and represents an essential *in vivo* process mediating proper vascular function. During atherosclerotic plaque formation, basal autophagy is stimulated in all major cell types, including macrophages, to blunt inflammatory signaling and to suppress atheroprogression (De Meyer et al., 2015). Indeed, disruption of essential autophagy-related genes (Atg5, Atg7), either in macrophages or VSMCs, accelerates atherosclerotic plaque development in mice (Liao et al., 2012; Razani et al., 2012; Grootaert et al., 2015; Osonoi et al., 2018). Macrophage autophagy not only protects against apoptosis, it also plays a prominent role in the clearance of apoptotic cells by efferocytosis (Liu et al., 2014). Indeed, Martinez et al. (2011) described engagement of MAPLC3A (LC3)-associated phagocytosis (LAP), which is distinct from the classical autophagy pathway but is dependent upon autophagy-specific genes such as Beclin1, Atg5 and Atg7. LAP is required for the engulfment and degradation of apoptotic cells. As a consequence, plaques of macrophage-specific Atg5-knockout mice reveal increased apoptosis, larger necrotic cores and overall lesion complexity (Liao et al., 2012). Moreover, defective autophagy in plaque macrophages is associated with proatherogenic inflammasome activation (Razani et al., 2012). This effect is probably mediated by crystalline cholesterol that accumulates in plaques with a macrophage-specific Atg5 deletion, and/or by the inefficient removal of damaged mitochondria and subsequent superoxide/ROS production (Razani et al., 2012). Additional *in vitro* experiments have shown that macrophage autophagy promotes reverse cholesterol transport and regulates the delivery of lipid droplets to lysosomes in macrophage foam cells, where lysosomal acid lipase-dependent lipolysis leads to the generation of free cholesterol for efflux (Ouimet et al., 2011). In this way, macrophage autophagy suppresses foam cell formation and contributes to the regression of atherosclerotic plaques. Several lines of evidence indicate that the uptake and degradation of cellular cargo in lysosomes, a process known as autophagic flux, is functional and atheroprotective during early atherosclerosis, but is stalled or becomes defective in macrophages of advanced atherosclerotic plaques (De Meyer et al., 2015). The underlying mechanisms are poorly defined, but may include (1) deposition of ceroid, an insoluble and indigestible complex of proteins and oxidized lipids that accumulates in lysosomes, (2) lysosomal dysfunction through permeabilization of the lysosomal membrane and/or the accumulation of lipids, and (3) age-dependent inhibitory effects (Kurz et al., 2007). These findings highlight the need for pharmacological interventions with compounds that stimulate the protective effects of autophagy in the atherosclerotic plaque. Many pharmacological approaches for the induction of macrophage autophagy in atherosclerosis have been proposed (Maiuri et al., 2013; Sergin and Razani, 2014; De Meyer et al., 2015). One straightforward and exciting pathway for maintaining sufficiently high levels of macrophage autophagy is by augmenting transcription factor EB (TFEB), a recently identified master regulator of autophagic activity and lysosome biogenesis. By overexpressing TFEB in macrophages, autophagy-lysosomal dysfunction is restored, which translates into broad atheroprotection (Sergin et al., 2017; Evans et al., 2018). TFEB also stimulates genes involved in endocytosis and phagocytosis. As mentioned above, these processes may help to reduce lesion complexity by clearing apoptotic cells via efferocytosis. Interestingly, TFEB overexpression can be pharmacologically induced by treating mice with the natural occurring disaccharide trehalose (Sergin et al., 2017; Evans et al., 2018), although the precise mechanism by which this occurs is unknown. It is hypothesized that trehalose is taken up by fluid-phase endocytosis and accumulates in the endosome-lysosomal system where it modulates its function. Of note, given the large amounts of trehalase in the gastrointestinal tract, oral dosing of trehalose should be avoided (Sergin et al., 2017; Evans et al., 2018). In future studies, it would be recommendable to use degradation-resistant trehalose analogs or to co-administer trehalase inhibitors. Another similar approach for inducing macrophage autophagy is the activation of nuclear factor (erythroid-derived 2)-like 2 (Nrf2) (Lazaro et al., 2018). This transcription factor is responsible for regulating a broad network of cytoprotective and antioxidant genes, including genes that are part of the autophagy machinery (e.g., SQSTM1/p62, Atg5). Treatment of ApoE^-/-^ mice with the Nrf2 inducer tert-butyl hydroquinone (tBHQ) provides atheroprotective effects, not only through the concerted upregulation of antioxidant and anti-inflammatory mechanisms, but also by enhancing autophagic flux in the vessel wall (Lazaro et al., 2018). Apart from trehalose, several other natural products are able to stimulate autophagy in macrophages including adenosine derivative cordycepin (Li et al., 2017), ursolic acid (Leng et al., 2016) or ginsenoside Rb1 (Qiao et al., 2017), the most abundant active component of ginseng. All these compounds attenuate atherogenesis or promote plaque stability in mice via the activation of macrophage autophagy. Recently, microRNA-33 (miR-33) has been found to suppress macrophage autophagy in atherosclerosis (Ouimet et al., 2017). Inhibition of miR-33 with anti-miR-33 increases expression of its direct target genes in the autophagy pathway (i.e., Atg5, Lamp1, and Prkaa1 in mice), but also promotes AMPK-dependent activation of the FOXO3 and TFEB transcription factors. In this way, treatment of atherosclerotic LDLR^-/-^ mice with anti-miR-33 restores defective autophagy in plaque macrophages, increases lysosomal biogenesis and triggers efferocytosis via an autophagy-dependent mechanism to reduce plaque necrosis (Ouimet et al., 2017). Other approaches that are worthwhile to mention include stimulation of macrophage autophagy via mild induction of ER stress (Ma et al., 2016) and statin therapy, even though the latter strategy is controversial. Some groups claim that statins block the maturation of autophagosomes, even with submicromolar statin concentrations (Miettinen and Bjorklund, 2015), which leads to a reduced basal autophagic flux. This block in autophagosome maturation increases the levels of autophagic markers, which might lead to the incorrect conclusion that statins increase autophagy (Miettinen and Bjorklund, 2016). In this light, it is tempting to speculate that reduced autophagic flux caused by the administration of statins may function as a mechanism for muscle toxicity and statin-induced myopathies (Miettinen and Bjorklund, 2016), which are well-known adverse effects of statin use. However, despite the abovementioned assumptions, growing evidence indicates that cholesterol depletion (via statins or other compounds) induces autophagy (Cheng et al., 2006) and that statin therapy attenuates pro-inflammatory effects and cholesterol accumulation in macrophages via autophagy-dependent signaling pathways (Huang et al., 2015; Han et al., 2018).

Although autophagy is an important subcellular pathway that mediates macrophage survival (Grootaert et al., 2018), exuberant induction of autophagy can stimulate macrophage death via the poorly understood type II programmed cell death, also termed autosis (Liu and Levine, 2015). In atherosclerotic plaques, selective induction of macrophage autosis may occur after stent-based delivery of rapamycin-derivatives (rapalogs) such as everolimus, and leads to a marked reduction of macrophages without altering the plaque VSMC content (Martinet et al., 2007b; Verheye et al., 2007). Rapamycin or rapalogs stimulate autophagy through inhibition of mTOR, a serine/threonine protein kinase that controls a variety of cellular functions including protein translation and cell proliferation. Knockdown of mTOR by mTOR-specific siRNA clears macrophages in a similar way (i.e., via induction of selective autophagy-mediated macrophage death) and inhibits the progression and destabilization of atherosclerotic plaques by downregulating MMP2 and MCP1 expression and by increasing the thickness of the fibrous cap (Wang et al., 2013; Zhai et al., 2014). It has been proposed that autophagy-mediated cell death using high concentrations (μM range) of rapamycin (or a rapalog), unlike apoptosis or necrosis, is the preferred type of death for selective depletion of macrophages in atherosclerotic plaques because macrophages undergoing this type of death literally digest themselves to death, without any major release of cellular content that could evoke an inflammatory response following post-autophagic necrosis (Martinet et al., 2007c). However, more recent studies showed that everolimus-treated macrophages secrete pro-inflammatory cytokines (e.g., IL-6, TNFα) and chemokines (e.g., MCP1) prior to autosis, a phenomenon that is not autophagy-dependent, but mediated through activation of p38 MAP kinase (Martinet et al., 2012). This finding indicates that everolimus-induced macrophage death is not a harmless event and provides a rationale for combined treatment of atherosclerotic plaques with everolimus and an anti-inflammatory agent (e.g., glucocorticoid) that suppresses inflammatory responses without affecting the ability of everolimus to deplete macrophages in atherosclerotic plaques (Martinet et al., 2012). Interestingly, a recent report showed that relatively low levels of rapamycin (50–100 nM) do not induce macrophage autosis, but facilitate autophagic removal of LPS-induced pro-IL-1β protein and mitochondrial ROS, thereby inhibiting activation of p38 MAP kinase and NF-κB, which in turn downregulates secretion of several pro-inflammatory cytokines including IL-6, IL-8, and MCP1 (Ko et al., 2017). In addition, low levels of rapamycin cause transcriptional upregulation of SQSTM1/p62, which potentiates autophagy and activates the Nrf2 pathway to further suppress mitochondrial ROS (Ko et al., 2017). Overall, these findings suggest that the final concentration of rapamycin (or rapalogs), used either *in vitro* or *in vivo*, is an important factor that determines macrophage fate (autosis or survival) and the cellular pro-inflammatory phenotype.

## Interrelationship Between Cell Death Pathways and Concluding Remarks

Research in the past two decades has demonstrated that macrophage death is critically involved in the formation and destabilization of atherosclerotic plaques. The use of pharmacological compounds modulating macrophage death is beneficial not only in preventing atherogenesis, but also in promoting plaque stability and even regression of established plaques. However, the question remains whether we should induce or prevent macrophage death and in which stage of the plaque. Preventing cell death is technically more challenging than the induction of cell death (Hotchkiss et al., 2009). Different forms of cell death can occur simultaneously, particularly in advanced atherosclerotic plaques, because of the coordinated action of multiple death-inducing stimuli. Accordingly, it may be necessary to target multiple death pathways. Another aspect that complicates therapeutic inhibition of cell death is the crosstalk between cell death mechanisms (Nikoletopoulou et al., 2013). Apoptosis, autophagy, and (regulated) necrosis were initially considered to be mutually exclusive states. However, recent findings reveal a balanced interplay between these types of death so that blocking one type of death may sensitize cells to initiate another death pathway. For example, inhibition of caspases by the pan-caspase-inhibitor zVAD is sufficient to prevent apoptosis in many experimental models, but may facilitate the necroptosis program downstream of TNFR. Accordingly, “Death by any other name” (referring to the novel by Daphne Kapsali) as well as “Dosis sola facit venenum” (referring to Paracelsus) seem to be very important issues when targeting macrophage death in atherosclerotic plaques. In line with these findings, several effector molecules and signaling pathways have been identified as key mediators in different types of cell death (Chen et al., 2018).

Macrophage-specific initiation of cell death can have plaque-stabilizing effects. However, the type and timing of cell death induction might be important. Indeed, one must be careful because induction of macrophage apoptosis in plaques with impaired efferocytosis, such as in late stages of atherosclerosis, represents a certain risk. In contrast, pharmacological inhibition of necrosis improves several features of plaque stability such as lowering plaque inflammation, reducing oxidative stress, and increasing collagen content and fibrous cap thickness. Moreover, inhibition of macrophage necroptosis could be a promising therapeutic strategy to prevent the development of a vulnerable plaque. Interfering with other types of regulated necrosis in macrophages, such as pyroptosis, ferroptosis, and parthanatos is not clear-cut at present and deserves further investigation. Last but not least, autophagic flux becomes defective in macrophages of advanced atherosclerotic plaques. A growing body of evidence indicates that treatment with an autophagy inducer can be exploited as a potential strategy to prevent plaque formation and destabilization. Of note, there is currently a strong scientific rationale for recommending combination therapy to treat or prevent atherosclerosis. Indeed, inhibiting various types of cell death simultaneously via combined therapy could be an important emerging concept in the field of atherosclerosis, yet very little experimental evidence exists that supports this approach. In our opinion, treatment with an autophagy inducer is one of the most interesting strategies to stabilize vulnerable plaques, on top of statin therapy (or other lipid-lowering interventions), for several reasons. By clearing damaged organelles and misfolded proteins, autophagy acts as a cellular safeguard that (i) protects vascular cells against apoptosis, (ii) plays a prominent role in the clearance of apoptotic cells by efferocytosis and the prevention of (secondary) necrosis, (iii) blocks inflammation and (iv) enhances cholesterol efflux from macrophage-derived foam cells in different stages of atherosclerosis. Thus by stimulating autophagy, several cell death pathways are impaired. However, inducing autophagy in macrophages to an extent that leads to autophagy-induced death (autosis) might be detrimental due to induction of an inflammatory response. Moderate stimulation rather than excessive stimulation of autophagy is recommended and this issue needs more attention in future studies. It is also noteworthy that besides standard therapy (use of small organic molecules or biological agents such as antibodies) long non-coding RNAs (lncRNAs) and miRNAs are gaining more and more importance in cell death research and clearly affect different types of macrophage death in atherosclerosis. Indeed, recent evidence indicates that macrophage apoptosis is regulated by exosomal lncRNA growth arrest specific transcript 5 (GAS5) (Chen et al., 2017) and several miRNAs including miR-21 and miR-30c-5p (Canfran-Duque et al., 2017; Ceolotto et al., 2017). The latter also modulates pyroptosis (Li et al., 2018), while other LncRNAs represent key regulating factors during efferocytosis or autophagic flux (Guo et al., 2019; Ye et al., 2019). Although these findings open interesting therapeutic perspectives, several limitations and challenges such as target specificity, route of delivery and stability, should be resolved before these RNA drugs can reach clinical applications.

## Author Contributions

IC and PP have made the figures. All authors contributed to the writing of the manuscript.

## Conflict of Interest Statement

The authors declare that the research was conducted in the absence of any commercial or financial relationships that could be construed as a potential conflict of interest.

## Footnotes

1 https://clinicaltrials.gov
